# Congenital toxoplasmosis in Austria: Prenatal screening for prevention is cost-saving

**DOI:** 10.1371/journal.pntd.0005648

**Published:** 2017-07-10

**Authors:** Andrea-Romana Prusa, David C. Kasper, Larry Sawers, Evelyn Walter, Michael Hayde, Eileen Stillwaggon

**Affiliations:** 1 Department of Pediatrics and Adolescent Medicine, Toxoplasmosis Reference Laboratory, Medical University of Vienna, Vienna, Austria; 2 Department of Laboratory Medicine, Medical University of Vienna, Vienna, Austria; 3 Department of Economics, American University, Washington DC, United States of America; 4 Institute for Pharmaeconomic Research, Vienna, Austria; 5 Department of Economics, Gettysburg College, Gettysburg, Pennsylvania, United States of America; Johns Hopkins University, UNITED STATES

## Abstract

**Background:**

Primary infection of *Toxoplasma gondii* during pregnancy can be transmitted to the unborn child and may have serious consequences, including retinochoroiditis, hydrocephaly, cerebral calcifications, encephalitis, splenomegaly, hearing loss, blindness, and death. Austria, a country with moderate seroprevalence, instituted mandatory prenatal screening for toxoplasma infection to minimize the effects of congenital transmission. This work compares the societal costs of congenital toxoplasmosis under the Austrian national prenatal screening program with the societal costs that would have occurred in a No-Screening scenario.

**Methodology/Principal findings:**

We retrospectively investigated data from the Austrian Toxoplasmosis Register for birth cohorts from 1992 to 2008, including pediatric long-term follow-up until May 2013. We constructed a decision-analytic model to compare lifetime societal costs of prenatal screening with lifetime societal costs estimated in a No-Screening scenario. We included costs of treatment, lifetime care, accommodation of injuries, loss of life, and lost earnings that would have occurred in a No-Screening scenario and compared them with the actual costs of screening, treatment, lifetime care, accommodation, loss of life, and lost earnings. We replicated that analysis excluding loss of life and lost earnings to estimate the budgetary impact alone.

Our model calculated total lifetime costs of €103 per birth under prenatal screening as carried out in Austria, saving €323 per birth compared with No-Screening. Without screening and treatment, lifetime societal costs for all affected children would have been €35 million per year; the implementation costs of the Austrian program are less than €2 million per year. Calculating only the budgetary impact, the national program was still cost-saving by more than €15 million per year and saved €258 million in 17 years.

**Conclusions/Significance:**

Cost savings under a national program of prenatal screening for toxoplasma infection and treatment are outstanding. Our results are of relevance for health care providers by supplying economic data based on a unique national dataset including long-term follow-up of affected infants.

## Author summary

*Toxoplasma gondii* is a widespread parasitic disease. In the event of primary infection during pregnancy, this parasite can be transmitted from mother to unborn child. Clinical presentation of congenital toxoplasmosis varies from asymptomatic to life-threatening risk for the fetus and infant and in later life. Prevention programs and screening strategies of health care providers vary in different countries. Austria has implemented mandatory prenatal screening for toxoplasmosis for four decades. The screening is free of charge for families and costs are covered by national health care providers. Compliance with the national program is good and outcomes for infected pregnant women and their infants since 1992 are well documented. We compared lifetime costs of screening, treatment, and follow-up with costs in a No-Screening scenario in an economic decision-analytic model. Prenatal screening resulted in substantial cost savings due to reduction in congenital toxoplasmosis and consequent injuries in affected children.

## Introduction

*Toxoplasma gondii* (*T*. *gondii*) is a protozoal parasite that infects up to 30% of humans globally, although prevalence of infection varies widely, from 10% to 80%, among world regions and within regions [1]. While the definitive host is the cat, sources of infection for humans include food, the water supply, and organ transplants as well as direct contact with cat feces in the soil and domestic litter [1–5]. Additionally and of particular concern is maternofetal transmission during pregnancy after primary infection.

Prevalence is high in South America and tropical Africa (>50%) [6], moderate in parts of western, central, and southern Europe (30% to 50%), and relatively low (10% to 30%) in northern Europe, North America, Southeast Asia, and the Sahara [7,8]. Prevention entails adequate cooking of meat and washing of fruits and vegetables as well as drinking water free of contamination with oocysts. Educational programs for prevention, however, can only reduce infection rates, not eliminate new infections, because most people, even those who are aware of the infection routes, do not know the source of their infection [3,4,6]. Most people infected postnatally have no recognized symptoms, but immune suppression due to medical conditions or treatments can lead to serious damage to the brain and eyes as a consequence of *T*. *gondii* infection. Infection with *T*. *gondii* that occurs during pregnancy can be transmitted to the unborn child and may have serious consequences, before or after birth, even in apparently asymptomatic infected newborns [9–11]. Three European countries—Austria, France, and Slovenia—have instituted mandatory prenatal screening for primary infections of *T*. *gondii* to minimize the harmful effects of infection on infants. This is the first systematic study of the cost of a European national prenatal screening program to reduce congenital toxoplasmosis (CT) and its sequelae [12].

### Congenital toxoplasmosis, risk of maternofetal transmission, and clinical manifestations

Women with primary infection with *T*. *gondii* during pregnancy may exhibit no symptoms, but there is about a 50% risk of transmission to the fetus and the possibility of mild to profound injury to the unborn child in untreated women [1]. The risk of maternofetal transmission increases over the course of the pregnancy, from very low risk in the first trimester to nearly 100% in the final weeks of pregnancy. In the event of transmission, risk of injury to the fetus varies inversely with gestational age, with the risk of profound injury greatest in the first trimester and the possibility of mild disease or no recognized symptoms in later stages of gestation [1,6,13,14].

Consequences of CT can include retinochoroiditis, hydrocephaly, cerebral calcifications, splenomegaly, hearing loss, blindness, and death [1,6,15,16]. In countries with prenatal screening for primary infections and consequent pre- and postnatal treatment, rates of CT and severity of symptoms in infants are lower than in countries without screening programs or compared to historical data before screening was initiated [7,10,17,18]. In comparison, a recent study of children in the United States with CT who had no pre- or postnatal treatment found that 91% of the children referred had visual and/or mental impairment by age 12 [9].

The risk of CT is complicated, however, by the diversity of genotypes of *T*. *gondii*. Type II predominates in Europe and was thought to be the predominant genotype in North America [6,19–21]. Recent research has identified greater diversity in US wild and domestic animals than was previously thought [22–24]. Types I and III and atypical genotypes are more common in Central and South America [25–27]. These latter strains are more virulent and are associated with ocular disease even when acquired postnatally by immune-competent persons [28]. South American genotypes are also associated with more serious injuries in CT [19,20,28–30].

### Prevalence and incidence in Europe and prevention programs

Prevalence of infection with *T*. *gondii* varies considerably in Europe, from 7% in Norway [31], 10% in the United Kingdom [32], 19% in Italy [33], 32% in Spain [34], 33% in Austria [31,35,36], and 34% in Slovenia [37], to 37 to 44% in France [7,38] (all reported since 2000). Over the past 20 years, prevalence has fallen rather dramatically in most of the high prevalence countries coincident with national education campaigns, which have perhaps led to changes in food preparation [7,31]. Systematic screening of pregnant women also plays an educational role in highlighting the importance of food safety and hygiene for the health of the unborn.

Countries with high prevalence in the past similarly had high rates of primary infection in women during pregnancy. This may seem paradoxical since the higher the prevalence among women of child-bearing age, the higher will be the proportion of women entering pregnancy who are immune. Since prevalence, however, increases with age, the majority of young women are not immune and continue to be at risk, presumably with the same food preparation habits as before.

The substantial drop in prevalence from the 1990s to the present was accompanied by a substantial drop in maternal incidence after an initial rise [7,17]. Austria in 1974, France in 1992, and Slovenia in 1995 initiated mandatory prenatal screening programs aimed at reducing maternofetal transmission as well as the severity of injury from CT. Numerous studies have reported that systematic prenatal screening and treatment were coincident with substantial reductions in maternofetal transmission and sequelae of CT [7,10,12,13,17,18,36,37,39–45].

No systematic economic evaluation of those programs, however, has been published. The countries with systematic prenatal screening and treatment programs face the paradox of successful prevention. Now there are so few children with serious, disabling symptoms of CT that it can appear that the risk of maternal infection does not warrant the expenditure for universal prenatal screening programs. Health budgets are under continual scrutiny. In some countries political currents have changed and the assumption of state responsibility for health is questioned. Moreover, there are diverse stakeholders in the decision to allocate funds to prenatal screening or to other national health needs: the Ministry of Health, insurance funds, the Ministry of Education, social security administrations, and families of affected children.

The purpose of the current work is to compare the societal costs of CT under the Austrian national program of prenatal screening for primary toxoplasmosis with the societal costs that would have occurred in the absence of the screening program.

### The Austrian national program of prenatal screening

In 1961, Thalhammer revealed a rate of CT of 78 per 10,000 live births for the Austrian population [46]. In response, mandatory prenatal screening for toxoplasma infection for all pregnant women was implemented in 1974 under the auspices of the national health care system [46,47]. This prenatal screening is part of a national prevention program called “Mother-Child-Booklet-Program” for all pregnant women and their infants through early childhood. The costs are covered by the government and the local health insurance funds; the program is free of charge for families.

The Austrian national program is described in detail in previous works [12,31,48]. Serological prenatal screening is performed ideally on a bimonthly schedule, at 8, 16, 24, and 32 weeks of gestation as well as a maternal or neonatal test for women seronegative up to the time of birth and women who have not been tested during pregnancy. In women with proven seropositivity before the current pregnancy, no further toxoplasma testing is necessary. Women who are tested and found to have been seropositive before conception require only one test. Those with suspected primary infection during pregnancy are tested twice. In Austria during this screening program, the local laboratories used 9 different test methods for the detection of IgM Toxo antibodies, each performed according to manufacturer recommendations. In the case of primary infection in a pregnant woman or to clarify suspicious test results, blood samples were retested in the reference laboratory. The Toxoplasmosis Laboratory at the Medical University of Vienna routinely uses the in-house Sabin Feldman dye test, immunosorbent agglutination assay (ISAGA)-IgM (bioMérieux, France), VIDAS Toxo IgG Avidity (bioMérieux, Frankreich), and PCR diagnostics for the detection of toxoplasma infections in pregnant women and their children.

In women with primary infection, amniocentesis and polymerase chain reaction of the amniotic fluid is recommended, but costs for those additional tests are not covered by the program. A positive result from amniocentesis identifies an affected fetus prenatally and influences the treatment during pregnancy. The routine PCR analysis used for the B1 gene after amniocentesis showed a sensitivity and specificity of 87.2% and 99.7%. Furthermore, the results revealed a positive predictive value and negative predictive value of 94.4% and 99.3% [48]. More recently, using the 529-bp PCR protocol improved sensitivity up to 100.0% [49].

Pregnant women are treated after the diagnosis of primary infection until birth, and infants with proven or suspected congenital infection are treated during the first year of life. In cases of CT, additional investigation, including cranial ultrasound, funduscopy, and complete blood count, is part of the program. The screening program reached 93% of pregnant women over the period covered by this analysis, although the ideal schedule was not achieved for most women [31].

### The Austrian Toxoplasmosis Register

The Austrian Toxoplasmosis Register records the serology history and birth outcomes for 1,387,680 pregnant women from 1992 to 2008 [12]. All cases of CT are recorded in the Register and thus it provides the basis for evaluating the costs of the program and pediatric outcomes over the 17-year period. In 10% of women no toxoplasma testing was necessary due to proven seropositivity before pregnancy. Screening confirmed additional infected women, resulting in seroprevalence of 34.4% used in the model [31]. The Register reported 70 women with primary infection of *T*. *gondii* and 8 cases of CT per year. The management of women and infants was stable, as was the rate of toxoplasma infection, during the observation period. Pediatric long-term follow-up revealed that 81% of infants with *T*. *gondii* infection did not show any clinical signs as of May 2013. All clinical variables for infection, transmission, and outcomes in infants are shown in Table 1.

**Table 1:** Probabilities.

Node	Test date	Clinical variable	Point estimate	References
1	*No Screening decision*			
2		Prob primary infection in pregnancy	0.000845	[12]
3		Prob fetal infection	0.508	[12]
4		Prob fetal death due to CT	0.05	[50,51]
5		Prob asymptomatic CT	0.06	[1]
5		Prob visual impairment in CT	0.48	[1,52]
7		Of which mild (severe)	0.09 (0.91)	[1,52]
5		Prob visual and cognitive impairment in CT	0.45	[1,52,53]
10		Of which mild (severe)	0.39 (0.61)	[1,52,53]
5		Prob visual, cognitive, hearing impairment, CT	0.01	[1,52,53]
1	*Screening decision*			
18	*8 Weeks*	Prob IgG(+) (maternal seroprevalence)	0.344	[31]
19		Prob IgG(+) IgM(+)	0.000845	[12]
20		Prob CT (maternofetal transmission)	0.034	[1,52–54]
21		Prob asymptomatic CT	0.58	[11,13,39,43–45,55–57]
21		Prob visual impairment in CT	0.3	[11,13,39,43–45,55–57]
21		Prob visual and cognitive impairment in CT	0.095	[11,13,39,43–45,55–57]
21		Prob visual, cognitive, hearing impairment, CT	0.005	[11,13,39,43–45,55–57]
21		Prob fetal death due to CT	0.02	[50]
29	*16 Weeks*	Prob IgG(+) (primary infection in pregnancy)	0.000845	[12]
30		Prob CT (maternofetal transmission)	0.17 [36 of 217]	[12]^a^
31		Prob asymptomatic CT	0.61 [22 of 36]	[12]^a^
31		Prob visual impairment in CT	0	[12]^a^
31		Prob visual, cognitive impairment CT (cerebral)	0.28 [10 of 36]	[12]^a^
34		Of which requiring special school	0.5 [5 of 10]	[12]^a^
31		Prob visual, cognitive, hearing impairment, CT	0	[12]^a^
31		Prob fetal death due to CT	0.11 [4 of 36]	[12]^a^
40	*24 Weeks*	Prob IgG(+) (primary infection in pregnancy)	0.000845	[12]
41		Prob CT (maternofetal transmission)	0.09 [36 of 398]	[12]^a^
42		Prob asymptomatic CT	0.86	[12]^a^
42		Prob visual impairment CT (1 w/ hearing loss)	0.056 [2 of 36]	[12]^a^
42		Prob visual and cognitive impairment in CT	0.056 [2 of 36]	[12]^a^
45		Of which cerebral toxoplasmosis	0.5 [1 of 2]	[12]^a^
45		Of which physical impairment	0.5 [1 of 2]	[12]^a^
42		Prob visual, cognitive, hearing impairment, CT	0	[12]^a^
42		Prob fetal death due to CT	0.028 [1 of 36]	[12]^a^
51	*32 Weeks*	Prob IgG(+) (primary infection in pregnancy)	0.000845	[12]
52		Prob CT (maternofetal transmission)	0.13 [48 of 368]	[12]^a^
53		Prob asymptomatic CT	0.79 [38 of 48]	[12]^a^
53		Prob visual impairment in CT	0.13 [6 of 48]	[12]^a^
53		Prob visual or cognitive impairment in CT	0.08 [4 of 48]	[12]^a^
56		Of which cerebral toxoplasmosis	0.75 [3 of 4]	[12]^a^
57		Of which requiring special school	0.33 [1 of 3]	[12]^a^
56		Of which physical impairment	0.25 [1 of 4]	[12]^a^
53		Prob visual, cognitive, hearing impairment, CT	0	[12]^a^
63		Prob no PCR of amniotic fluid	0.43	[12]^a^
66	*40 Weeks*	Prob IgG(+) (primary infection in pregnancy)	0.000845	[12]
67		Prob CT (maternofetal transmission)	0.37 [18 of 49]	[12]^a^
68		Prob asymptomatic CT	0.944 [17 of 18]	[12]^a^
68		Prob visual impairment in CT	0	[12]^a^
68		Prob visual and cognitive impairment in CT	0.056 [1 of 18]	[12]^a^
68		Prob visual, cognitive, hearing impairment, CT	0	[12]^a^

^a^ These data were obtained by the Austrian Toxoplasmosis Register. This register comprises all suspected infections with *T*. *gondii* during pregnancy and all congenitally infected infants in Austria since 1992.

## Method

We retrospectively analyze data from the Austrian Toxoplasmosis Register for birth cohorts from 1992 to 2008 and clinical data from pediatric long-term follow-up to May 2013 [12]. Data were recorded at the Medical University of Vienna, Austria, in coordination with local nurses, physicians, specialists, and medical care centers. Average annual number of births was 81,628 over the 17-year period [12] and 76,547 over the last decade (www.statistik.at). We compared societal costs of illness over the lifetimes of affected children of the Austrian national program as it was carried out with the lifetime societal costs estimated in the hypothetical scenario of Austria if it had not implemented prenatal screening in those years.

We use TreeAge Pro Suite 2015 software (TreeAge Software, Inc., Williamstown, MA, USA) to construct a decision-analytic model. Using a societal perspective, we include the costs of treatment, care, and accommodation of injuries projected over the lifetimes of affected children, and lost productivity that would have occurred in a No-Screening scenario with the actual costs of screening, treatment, projected lifetime care and accommodation, and lost productivity in Austria for all of the children in the Register from 1992 to 2013.

The current work follows a template established in a decision-analytic model for a hypothetical prenatal serologic screening program for the United States [51]. The current work is the first to use clinical data on specific child outcomes with local costs to calculate the lifetime costs and benefits of a mandatory national prenatal screening program as it has been carried out over time compared to the costs that would have occurred if there had been no screening program.

The model (decision tree) contains two kinds of variables: probabilities at chance nodes (circles) and costs of outcomes at terminal nodes (triangles). Clinical variables are listed in Table 1 and represent the chance of primary infection during pregnancy, fetal infection, and pediatric clinical long-term outcomes. For the No-Screening branch, probabilities are based on international experience reported in peer-reviewed literature and synthesized in the US model [51]. Because this is a retrospective study dating back to 1992, the use of historical data for the counterfactual No-Screening scenario is appropriate. The risk of fetal infection in the No-Screening scenario is taken from the actual rate of transmission among untreated women recorded in the Austrian Toxoplasmosis Register [12]. In the Screening branch, probabilities for results at the 8-week screening are also derived from the literature in [51] because the small number of cases in the Austrian Register makes a comparison unreliable. For all other branches of the Screening arm, the probabilities are calculated from the Austrian Toxoplasmosis Register and thus represent actual Austrian experience recorded by the Toxoplasmosis Laboratory at the Medical University of Vienna, Vienna, Austria [12].

Costs of serology, treatment, and lifetime costs of special care and lost productivity for affected infants and their parents are listed in Table 2. Costs of serology, other tests, and medications are derived from recorded expenses of the Austrian program from the years 1999–2013 and adjusted to 2012 prices. For test and medication costs we use the average of costs reported by health insurance funds. Lifetime costs of injuries of CT include treatment, accommodation, special schooling, loss of earnings for affected infants, and loss of parental earnings. Earnings are used as a proxy for the lifetime productivity that is lost by the family and the society for infants affected by CT and their parents. Estimates of costs are derived from the literature for Austria (adjusted to costs for 2012) and, when necessary, for neighboring countries (adjusted to Austrian costs for 2012). Direct costs and productivity losses are discounted annually at 3% for as long into the future as each cost occurs. Direct costs for medication represent average maternal and infant treatment costs. Costs of special treatment are assigned to the actual child outcomes in the Austrian Register [12]. Detailed explanation of cost derivation can be found in the methodological supplement, S1 Methods. For the cost assigned to death, *in utero* or neonatal, we derive a Value of Statistical Life (VSL) for Austria in 2012 using the recommendation of the OECD (Organization of European Cooperation and Development), which is based on a meta-analysis of more than 800 studies of VSL [58]. The background on the use of VSL and the derivation of our estimate for Austria are explained in the methodological supplement, S1 Methods.

**Table 2:** Direct and indirect costs.

Variable name	Description	Present value in euros (2012)	Source
*Test and medication costs*			
Fundus	Indirect funduscopy cost	54.26	a
InfIgGtest	Infant IgG test cost	13.48	b
InfIgMtest	Infant IgM test cost	17.69	b
MatIgGtest	Maternal IgG test cost	6.50	b
MatIgMtest	Maternal IgM test cost	6.89	b
PedCBC	CBC during pediatric treatment	24.85	c
PedConsult	Pediatric consultation cost*	0	a
PedCranialUltra	Pediatric cranial ultrasound cost	90.71	a
PedECG	Pediatric ECG cost	18.05	c
PedRx	Pediatric treatment costs 12 months	160.26	d
RxNegPCR	Maternal treatment costs with negative PCR of amniotic fluid	181.78	d
RxPosPCR	Maternal treatment costs with positive or no PCR of amniotic fluid	178.32	d
*Direct costs due to impairments*			
Cerebral	Treatment costs for cerebral CT	30,458	e, [59]
CognMild	Treatment costs for mild cognitive impairment	66,071	e, [59]
CognSevere	Treatment costs for severe cognitive impairment	445,536	e, [59]
HearingMild	Treatment costs for mild hearing impairment	20,924	e
SpecEdBlind	Special school costs for severe visual impairment	86,239	e, [60]
SpecEdMildCogn	Special school costs for mild cognitive impairment	73,699	e, [60]
SpecEdSevereCogn	Special school costs for severe cognitive impairment	713,141	e, [60]
VisualMild	Treatment costs for mild visual impairment	1,576	e, [60]
*Indirect costs due to impairments*			
ChildPyLoss	Productivity loss for severe cognitive impairment	561,721	e, [61,62]
ParentPyLoss	Productivity loss of parents	33,940	e, [60,63]
VisualSevere	Income loss and non-medical costs of severe visual impairment (for societal costs)	393,624	e, [64]
VisualSevere	Non-medical costs of severe visual impairment (for budget impact only)	327,594	e, [64]
VSL	Value of a statistical life	5.85 million	e, [58]
	Value of a statistical life (for budget impact only)	0	

* The standard pediatric protocol includes 5 well-baby consultations, so there is no additional expense for infants with CT.

Sources:

^a.^ Average for all 9 regional health insurance funds: Burgenländische Gebietskrankenkasse, Kärntner Gebietskrankenkasse, Niederösterreichische Gebietskrankenkasse, Oberösterreichische Gebietskrankenkasse, Salzburger Gebietskrankenkasse, Steiermärkische Gebietskrankenkasse, Tiroler Gebietskrankenkasse, Vorarlberger Gebietskrankenkasse, Wiener Gebietskrankenkasse;

^b.^ Average Austrian laboratory costs, numerous payers;

^c.^ BMGF, Austrian Federal Ministry of Health;

^d.^ Cost of treatment for health insurance funds;

^e.^ See methodology supplement, S1 Methods.

In addition to the costs that are assigned to each outcome as terminal nodes in the tree, we include the costs of amniocentesis with PCR, which is assigned to the group of women with primary infection. It is unnecessary to assign the costs to specific women because it does not change the overall costs. In Austria over the period, 60% of women with gestational toxoplasma infection underwent amniocentesis. The cost of PCR, which was €363.45, was absorbed by the local prenatal care centers. The total cost was almost €256,000. Since the decision tree calculates the cost per birth in the country, we assign the cost of PCR as overhead on all 1,387,680 births over the period. It is expected that the cost of PCR will drop significantly in the near future, to an estimated €100 when the testing is done routinely, reducing costs overall. Although the women and their insurers did not bear the cost, the expense does represent a societal cost and so we include it in the analysis.

**Fig 1:**
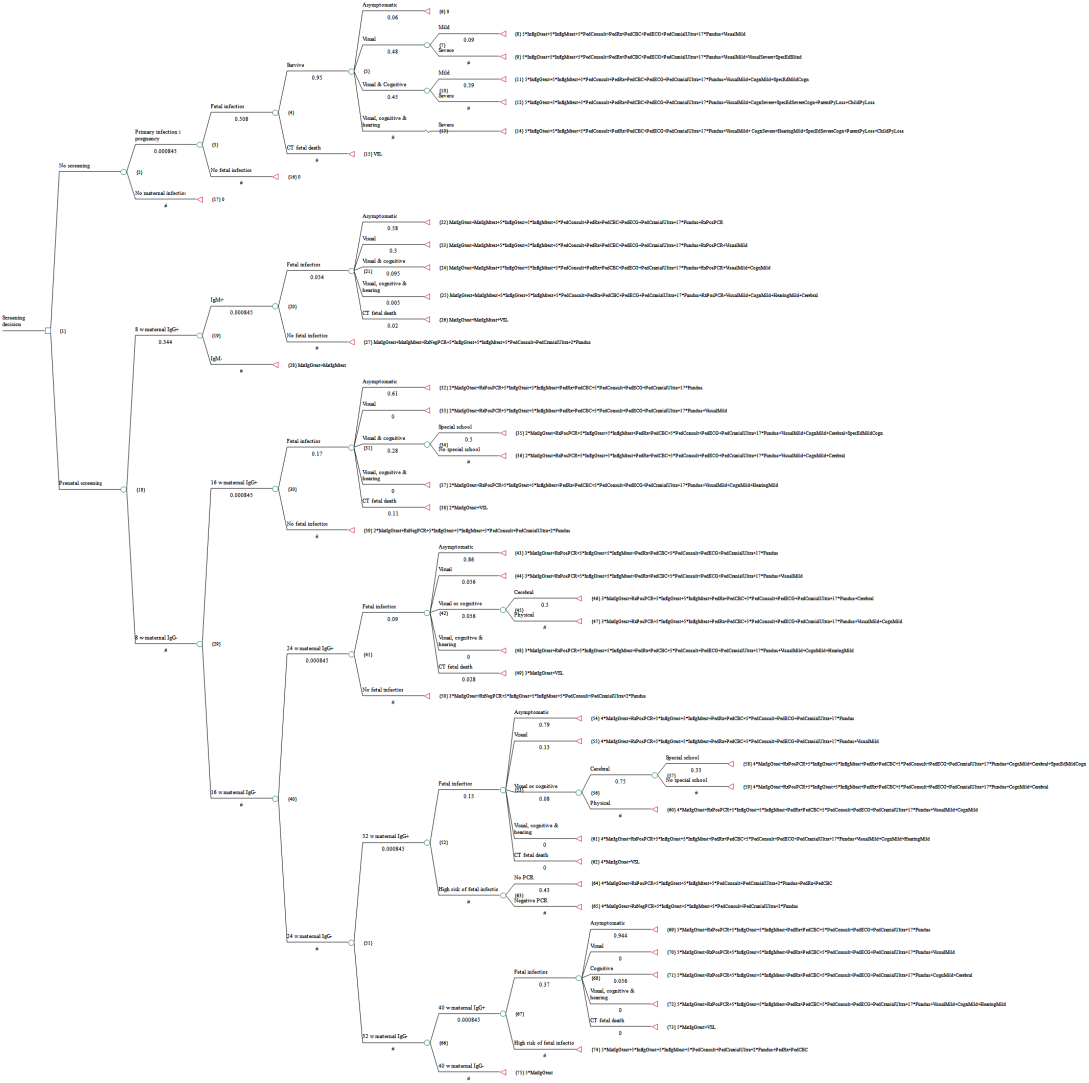
Decision tree before calculation. Tree with societal cost formulas at terminal nodes.

The decision tree shows the probabilities of all possible outcomes and the costs associated with each outcome. In Fig 1, each outcome has a conditional probability that is the product of the probabilities along each branch. The formulas at the terminal nodes for each outcome list the direct and indirect costs that are explained in Table 2.

The method outlined above is the conventional way to calculate the lower-cost option, including societal costs that are borne by affected infants, their families, and the economy as a whole, regardless of who pays. There could be times, however, that a Ministry of Health or other institution would like to know just the impact of a program on the government budget, not societal cost. For that reason, we also calculate the cost-saving option considering only those costs paid by government and public insurance funds, that is, omitting lost productivity of affected children and their parents and VSL.

To test the robustness of our results to variations in costs, we perform a sensitivity analysis using an Incremental Tornado diagram varying all costs –10% and +10%, except for test costs, which have a lower bound of €4, and VSL, which is given a range of €800,000 to €6,700,000. The former amount represents only the discounted valuation of productivity loss over the lifetime, and the latter amount is the upper bound of the OECD estimate of VSL. (See S1 Methods for explanation of VSL derivation.)

### Ethics statement

The maternal screening study was approved by the local ethics committee at the Medical University of Vienna, Vienna, Austria (824/2009). All adult subjects and parents of any child participants gave their informed consent orally in person or by telephone at the time of inclusion. The individuals were included in the nationwide toxoplasmosis register performed 1992‒2008 and their oral consent was documented in the register data file. Written consent could not be obtained, due to the fact that this was a nationwide study. The data were processed anonymously. The current economic study utilized anonymous data from the national screening program.

## Results

**Fig 2:**
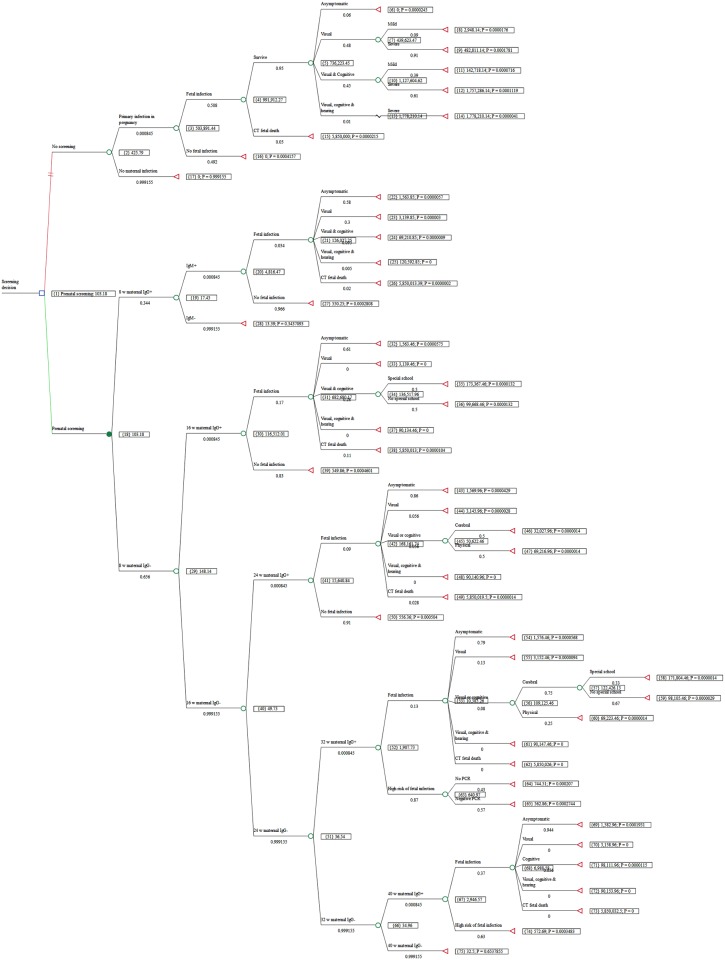
Decision tree after calculation. Tree showing results for societal costs.

In Austria, a country with a moderate seroprevalence of *T*. *gondii* during childbearing years, we recorded a total of 1,387,680 women giving birth between 1992 and 2008 (www.statistik.at). Fig 2 shows the decision tree after calculation of the lower-cost option, based on the probability of each outcome and the costs associated with each. As shown in Fig 2, and summarized in Table 3, lifetime societal costs of CT sequelae in the No-Screening scenario would have been €426 per birth, or about €35 million for all Austrian births in one year. Total societal costs in Austria that would have occurred without prenatal screening for nearly 1.4 million births over the 17 years would have been about €591 million, including costs for lifelong treatment and accommodation, as well as loss of earnings for affected children and their parents.

**Table 3:** Results.

	Societal costs (euros)			Budgetary costs (euros)		
Scenario	Cost per birth	Cost for all births in one year	Costs for all births 1992‒2008	Cost per birth	Cost for all births in one year	Costs for all births 1992‒2008
No Screening	426	34,756,486	590,860,267	219	17,905,970	304,401,485
Screening	103	8,422,401	143,180,822	33	2,711,690	46,098,730
Saving	323	26,334,085	447,679,445	186	15,194,280	258,302,755

In contrast, prenatal screening for toxoplasma infections according to the Austrian national program including costs of screening, maternal treatment, infant treatment, and lifetime costs for those infants with CT sequelae amounted to €103 per birth. The total cost of the Screening scenario, including lifetime costs of CT sequelae, was €8.4 million for all births in one year for Austria, or €143 million for 1.4 million births in the 17-year period.

As shown in Table 3, the prenatal screening option resulted in savings of €323 per birth, or about €26 million per year compared to No-Screening. For all births, screening saved about €448 million in 17 years.

Adding the cost of amniocentesis with PCR for 60% of the women with primary toxoplasma infection during pregnancy increased the cost per Austrian birth in the period by about €0.18, changing the difference in cost per birth of the entire screening and treatment program by a trivial amount.

### Actual program costs and the cost of CT for an affected child

The TreeAge program calculates all of the costs that occur in each scenario—the counterfactual (No screening) compared to all actual lifetime costs in Austria resulting under the screening scenario. Thus the TreeAge program attributes costs to the Screening scenario that result from treating infants who are infected despite the program, including those whose mothers were not screened or were screened inadequately, with the lifetime costs of follow-up, accommodation, and parental work time lost. In Austria, if there were no screening program, one must assume that the state would provide health care for a child born with, or who later develops, CT symptoms. So the costs of diagnosing and caring for a symptomatic infected child are not really costs of the screening program itself. They would occur (and in much larger numbers) without the national screening. The €8.4 million a year under the Screening scenario represents the costs of the screening program plus the lifetime societal cost for the affected children born during the 17-year period.

The screening program itself entails very little cost. It includes only testing all pregnant women (except those already known to be seropositive) and treating women with primary infection. It also would include the cost of treating the very few asymptomatic infected infants because without screening they would be missed, but with screening, they would be treated from birth. Under the screening program, there have been 70 incident infections in mothers per year. Without treatment, there would be a fetal infection rate of 0.508 [12] and a probability of asymptomatic CT of 0.06 [1]. Thus, there would be two asymptomatic infected newborns treated per year because of the screening program who would have been overlooked without screening (70 x 0.508 x 0.06 = 2.10). Costs for each of those children would be: 5 infant IgG test, 5 infant IgM test, pediatric treatment, CBC, ECG, cranial ultrasound, and 17 funduscopies, which amount to €1,372.

The costs of the screening program, shown in Table 4, total approximately €1.9 million per year for all pregnancies, or €25 per pregnancy. A new diagnostic appears likely with a test cost of about €4. Recalculating with a test cost of €4 would reduce the total cost of prenatal screening and maternal treatment from about €1.9 to about €1.2 million (calculation not shown).

**Table 4:** Annual cost of the screening program.

Category	Number affected	Unit cost euros (2012)	Total cost euros (2012)
Average annual number of pregnancies since 2000	76,547		
minus 10% of women already identified IgG^+^	7,655		
Women needing initial IgG	68,892	6.50	447,800
Women needing IgM (assuming 34% prevalence minus 10% identified)	18,370	6.89	126,570
Women IgG^‒^ needing 4 more tests	50,520	4 x 6.50	1,313,550
Primary infections requiring treatment	70	180	12,600
Amniocentesis cost	42	363.45	15,265
Asymptomatic CT newborns tested and treated	2	1,372	2,744
Total cost of prenatal screening and treatment	**€**1,918,529		

The costs of the screening program can be compared to the cost of caring for a child whose mother is not treated. The costs for individual services and productivity losses are listed in Table 2, but each symptomatic child generates multiple kinds of costs. In the tree before rollback (calculation), Fig 1, all the costs for an individual child for each outcome are listed at the terminal node. For example, in the No-Screening scenario, a child with severe visual, cognitive, and hearing impairment (Terminal node #14 in Fig 1) will incur the following costs (assuming symptoms at birth that lead to testing, treatment, and follow-up care): 5 infant IgG tests, 5 infant IgM tests, pediatric treatment, CBC, ECG, cranial ultrasound, and 17 funduscopies, as well as the direct costs and productivity losses for child and parents associated with severe visual, cognitive, and hearing impairment, and special education costs.

Fig 2 (Terminal node #14) shows the sum of those costs. The lifetime cost for one child with severe visual, cognitive, and hearing impairment is €1.8 million (€1,778,210). Thus the costs of the entire screening program for one year are nearly the same as the potential costs for a single severely affected child whose mother was not treated. A child with only severe visual impairment generates costs of €482,811 (at terminal node #9). The costs for four such children exceed the annual cost of the screening program. Without prenatal treatment, more than 90% of infected children have been found to have some form of serious impairment [1,9,52,53]. Prenatal screening with pre- and postnatal treatment as needed prevents or mitigates most injuries.

Austria has 70 primary infections per year [12]. If we assume 50% maternofetal transmission without prenatal treatment, as seen in Austrian women who were not treated [12], that would be 35 cases of CT each year, rather than the 8 cases per year under the treatment program, with symptoms ranging from mild visual impairment to fetal death. Because the model calculates costs on a population basis, the cost of €426 in the tree is a cost per Austrian birth, which is multiplied by the number of births, resulting in potential costs of €35 million for the 35 children who would be infected under the No-Screening scenario. The screening program costs €1.9 million per year while the societal costs of the No-Screening scenario are €35 million per year.

It is useful to see these costs in relation to overall Austrian government spending and Gross Domestic Product (GDP). The annual cost of the screening and treatment program is 0.007% of total Austrian public spending on health and 0.003% of overall Austrian government spending. The annual cost of the program is 0.0006% of Austrian GDP (Derived from data at www.focus-economics.com/country-indicator/austria/gdp-eur-bn; World Development Indicators, www.wdi.worldbank.org).

### Budget impact

**Fig 3:**
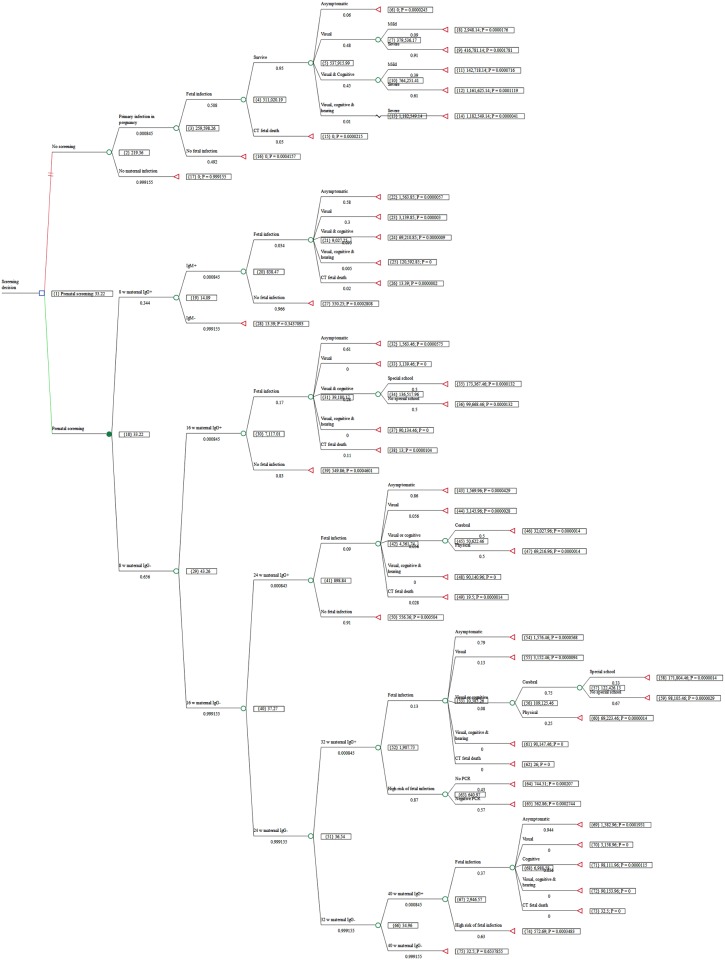
Decision tree for budget impact. Tree showing results, budget impact.

Calculating just the impact on the Austrian public budget—that is, omitting the lifetime costs of lost earnings that fall on affected children, their families, and society, and VSL for fetal and infant deaths, we find that the maternal screening program is still cost-saving. As seen in Fig 3, and summarized in Table 3, expenditures by government and government-sponsored insurers, based on Austrian experience over the period 1992 to 2008, cost €33 per birth compared to an estimated €219 per birth if the prenatal screening program had not been implemented in Austria. (As explained above, this overstates the budgetary cost of the screening program itself because it includes diagnosis and care of children who would be cared for under the Austrian health care system even without a screening program.) Even from the extremely narrow budgetary perspective, the Austrian national program has more than paid for itself in reducing the costs to the state and state-sponsored institutions of treating and educating children injured by CT by €186 per birth for 1.4 million births over the period. That amounts to a total budgetary saving of more than €258 million, or more than €15 million per year.

### Sensitivity analysis

**Fig 4:**
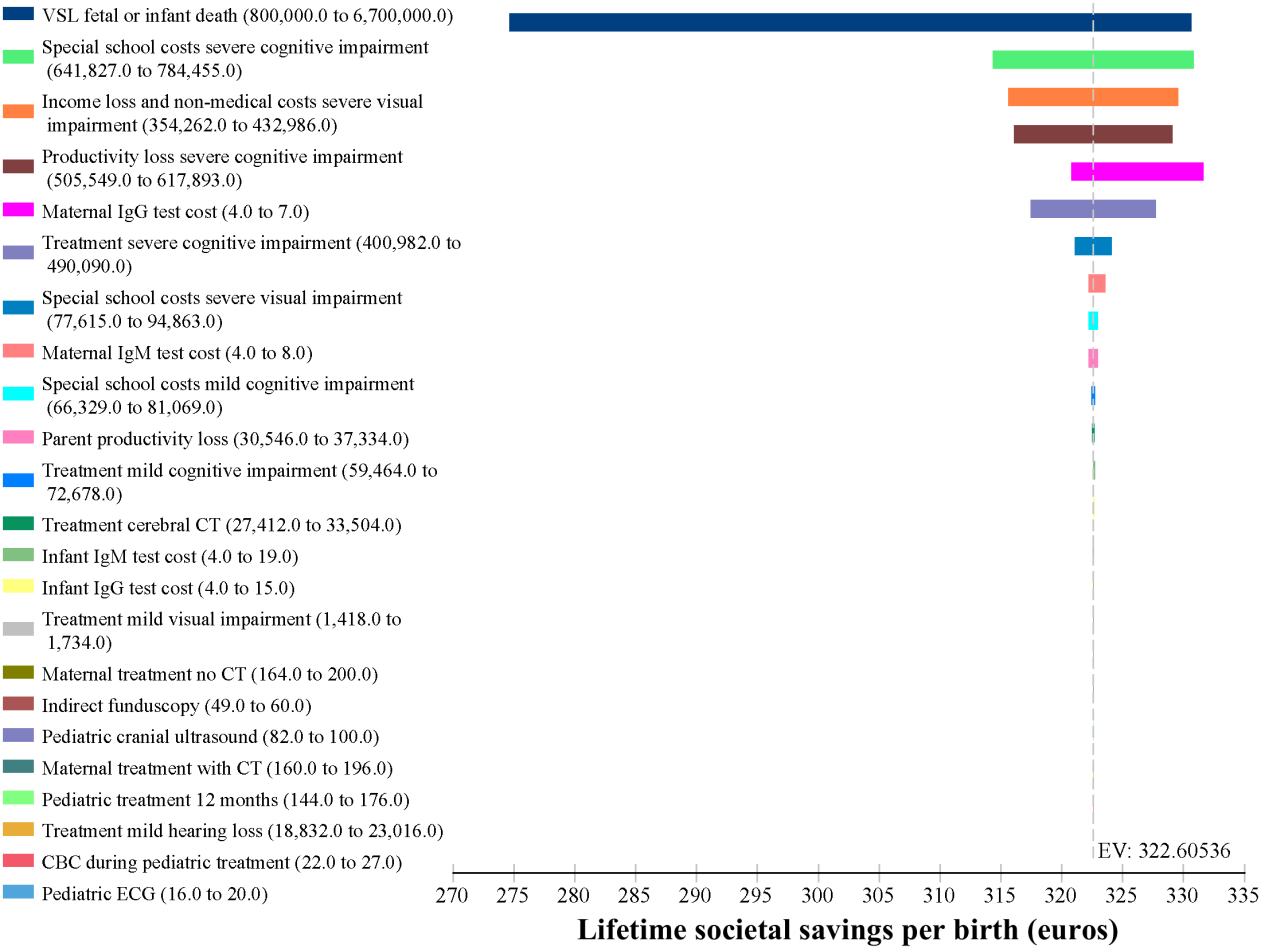
Incremental tornado sensitivity analysis, societal perspective.

**Fig 5:**
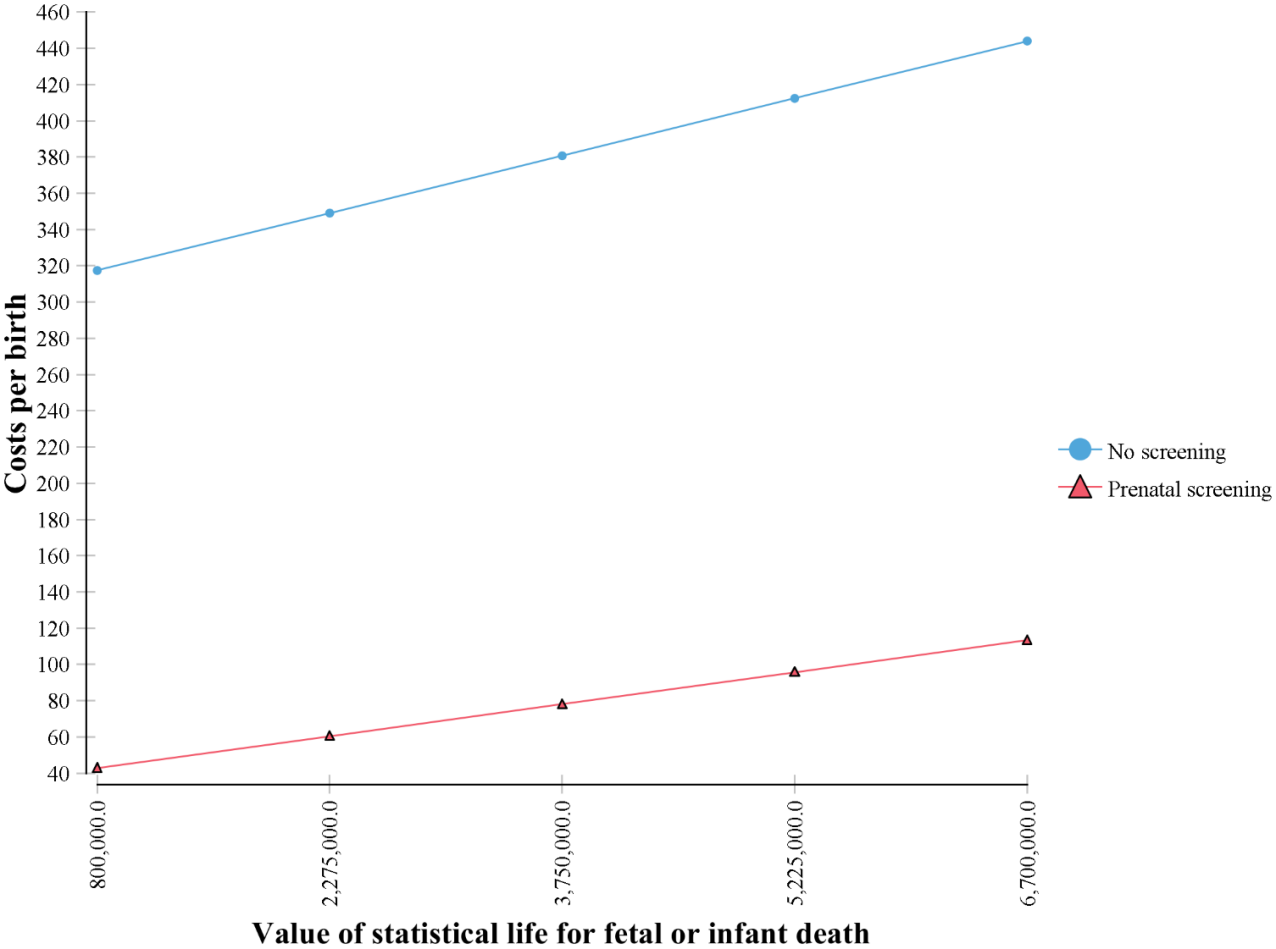
One-way sensitivity analysis on value of statistical life.

Results of the sensitivity analysis show that the savings both to society and to the government budget are robust to variations in all costs. Varying costs by ±10% had a trivial effect on cost per birth in the No-Screening and Screening scenarios and consequently on the savings that result from screening, for both the full societal cost and for the public budget. Fig 4 shows an Incremental Tornado Analysis from the societal perspective. The x axis shows the difference in costs per birth between the No-Screening and Screening scenarios with an Expected Value (EV) of €323. The horizontal bars show the full variation in the Expected Value (savings per birth) resulting from the range of values for each cost parameter. Both Fig 4 and Table 5 demonstrate the trivial impact on the large savings that result from screening. The variation in VSL had the greatest effect on costs, but even then the difference between low and high values for savings was only €56 and the savings from screening never fell below €275 per birth. Fig 5 shows the one-way sensitivity analysis on VSL in the societal model, which again demonstrates that whether one includes only the loss of earnings (€800,000) or the upper bound of the OECD estimate for VSL (€6.7 million), there is little impact on the savings derived from the screening program, showing the same minimum savings of €275 per birth seen in Fig 4 and Table 5.

**Table 5:** Incremental tornado risk report, societal perspective: Savings from screening.

Variable	Variable range (euros)	Savings from screening		
		Low (euros)	High (euros)	Spread (euros)
Value of statistical life for fetal or infant death	800,000 ‒ 6,700,000	274.61	330.68	56.07
Special school costs for severe cognitive impairment	641,827–784,455	314.33	330.88	16.55
Income loss and non-medical costs of severe visual impairment	354,262–432,986	315.59	329.62	14.02
Productivity loss for severe cognitive impairment	505,549–617,893	316.09	329.12	13.03
Maternal IgG test cost	4–7	320.80	331.66	10.86
Treatment costs for severe cognitive impairment	400,982–490,090	317.44	327.77	10.34
Special school costs for severe visual impairment	77,615–94,863	321.07	324.14	3.07
Maternal IgM test cost	4–8	322.22	323.60	1.38
Special school costs for mild cognitive impairment	66,329–81,069	322.19	323.03	0.84
Productivity loss of parents	30,546–37,334	322.21	323.00	0.79
Treatment costs for mild cognitive impairment	59,464–72,678	322.44	322.77	0.34
Treatment costs for cerebral CT	27,412–33,504	322.47	322.74	0.27
Infant IgM test cost	4–19	322.59	322.75	0.16
Infant IgG test cost	4–15	322.59	322.71	0.12
Treatment costs for mild visual impairment	1,418–1,734	322.55	322.66	0.11
Maternal treatment costs with no CT	164–200	322.58	322.63	0.05
Indirect funduscopy cost	49–60	322.58	322.63	0.05
Pediatric cranial ultrasound cost	82–100	322.59	322.62	0.04
Maternal treatment costs with CT	160–196	322.59	322.62	0.02
Pediatric treatment costs 12 months	144–176	322.60	322.61	0.02
Treatment costs for mild hearing impairment	18,832–23,016	322.60	322.61	0.02
CBC during pediatric treatment	22–27	322.60	322.61	0.00
Pediatric ECG cost	16–20	322.61	322.61	0.00

**Fig 6:**
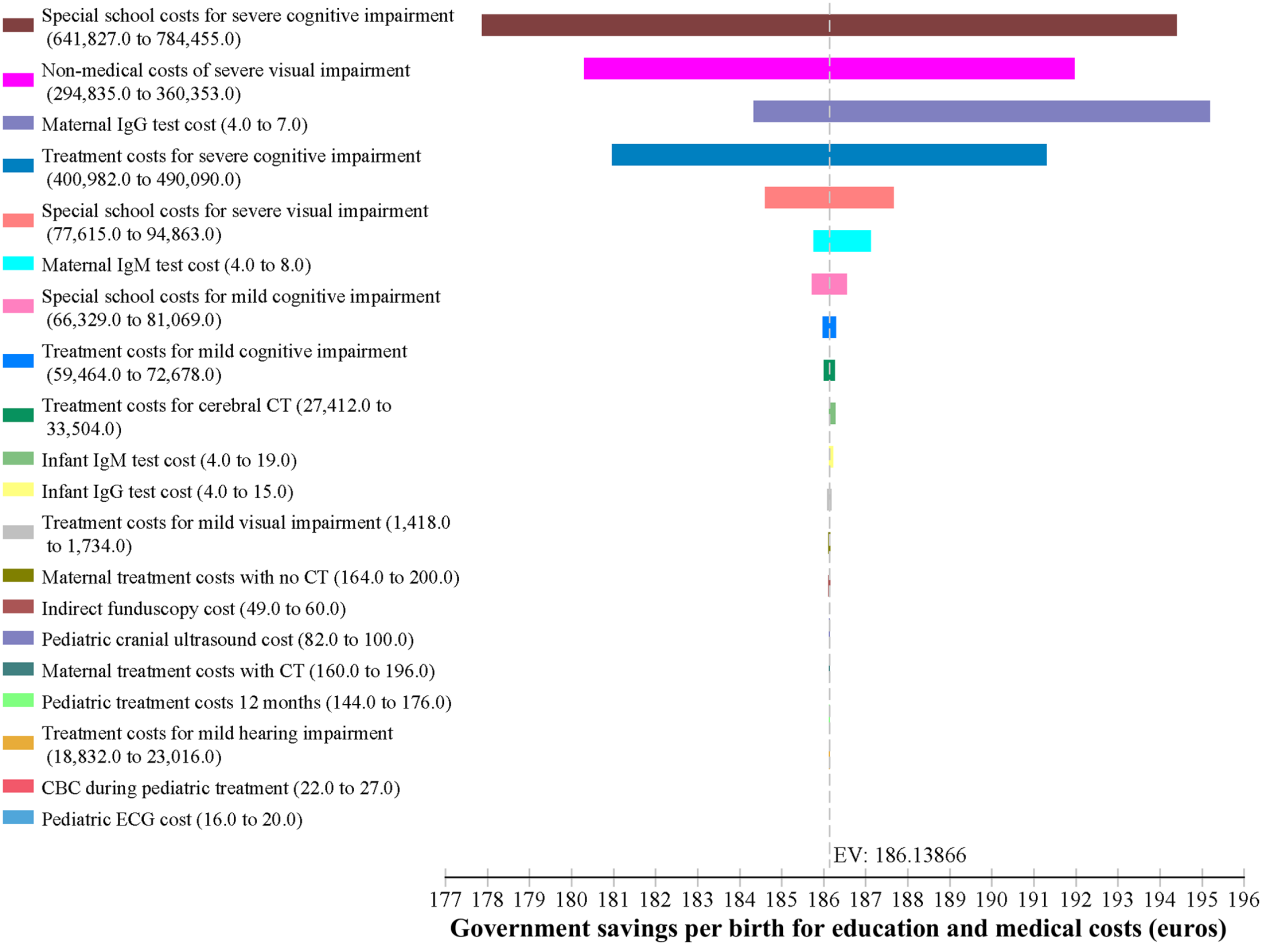
Incremental tornado sensitivity analysis (budget impact).

Fig 6 shows the Incremental Tornado Analysis for the Budget impact. The Expected Value, that is savings per birth, is €186. The variation in savings per birth never exceeds €17 and the minimum savings from the screening program for the budget is never less than €178 per birth, as seen also in Table 6.

**Table 6:** Incremental tornado risk report, budget impact: Savings from screening.

Variable	Variable range (euros)	Savings from screening		
		Low (euros)	High (euros)	Spread (euros)
Special school costs for severe cognitive impairment	641,827–784,455	177.86	194.41	16.55
Non-medical costs of severe visual impairment	294,835–360,353	180.30	191.97	11.67
Maternal IgG test cost	4–7	184.33	195.19	10.86
Treatment costs for severe cognitive impairment	400,982–490,090	180.97	191.31	10.34
Special school costs for severe visual impairment	77,615–94,863	184.60	187.67	3.07
Maternal IgM test cost	4–8	185.76	187.13	1.38
Special school costs for mild cognitive impairment	66,329–81,069	185.72	186.56	0.84
Treatment costs for mild cognitive impairment	59,464–72,678	185.97	186.31	0.34
Treatment costs for cerebral CT	27,412–33,504	186.01	186.27	0.27
Infant IgM test cost	4–19	186.12	186.28	0.16
Infant IgG test cost	4–15	186.12	186.24	0.12
Treatment costs for mild visual impairment	1,418–1,734	186.09	186.19	0.11
Maternal treatment costs with no CT	164–200	186.11	186.17	0.05
Indirect funduscopy cost	49–60	186.11	186.16	0.05
Pediatric cranial ultrasound cost	82–100	186.12	186.16	0.04
Maternal treatment costs with CT	160–196	186.13	186.15	0.02
Pediatric treatment costs 12 months	144–176	186.13	186.15	0.02
Treatment costs for mild hearing impairment	18,832–23,016	186.13	186.15	0.02
CBC during pediatric treatment	22–27	186.14	186.14	0.00
Pediatric ECG cost	16–20	186.14	186.14	0.00

## Discussion

In this retrospective study we compare the costs for a national program of prenatal screening for *T*. *gondii* with a No-Screening scenario for Austria, a country with moderate seroprevalence in women of childbearing-age and 1,387,680 births over the years 1992 to 2008. There have been few economic analyses of CT-prevention programs [51,65]. To our knowledge this is the first report of an economic decision-analytic model incorporating surveillance data from pregnancy through long-term pediatric follow-up for an entire nation over nearly two decades of observation. Thus our data are of special interest for physicians, health care providers, and policy makers in considering the implementation of a prevention program for CT.

The substantial reductions in primary infection, maternofetal transmission, and fetal and child injuries resulting from *T*. *gondii* infection during the implementation of the Austrian prenatal screening program from 1992 to 2008 have been reported elsewhere [12]. In the current work, our major finding demonstrates that a national program of prenatal screening and treatment to prevent congenital toxoplasmosis or reduce clinical symptoms in affected infants is cost-saving for governmental health care providers and for Austrian society. Under the Austrian national prenatal screening program, total societal savings are €323 per birth. Consequently, the screening program saved about €448 million in costs to Austrian society for the birth cohorts from 1992 to 2008. Even in narrowly budgetary terms, the prenatal screening program has saved the Ministry of Health, the Ministry of Education, and government-sponsored insurance funds €186 per birth, or more than €258 million over the period, averaging more than €15 million a year, because of injuries prevented in children of women with primary toxoplasma infection. Even large variations in all costs make little difference in results. This is not surprising given the profound injuries that can occur without treatment and the low cost of the intervention.

Even in a country where prevalence is falling due to greater awareness and success of primary prevention, prenatal screening is needed. Lower prevalence means that more women enter pregnancy susceptible to infection. Since seroprevalence increases with age, women in their childbearing years are among the vulnerable population that has grown over the past decades as prevalence has declined.

Under the Austrian national program of prenatal screening, there has been a dramatic reduction in maternofetal transmission of *T*. *gondii* and in the degree of injury in affected children compared to historical data before implementing the prenatal screening [46,47]. Interestingly, the Austrian Toxoplasmosis Register shows even greater success in child outcomes than observed in France even though the French protocol mandates monthly testing, compared to the Austrian program of bimonthly testing [12]. It seems, however, that in Austria, while women are attending prenatal checkups, most are not receiving the recommended number of blood tests for primary toxoplasma infection [12,31,35]. Education of women and obstetric staff should be a relatively inexpensive solution that would improve even further the success of the Austrian CT-prevention program and increase the cost saving beyond what we have measured based on actual experience.

Further examination of the Austrian data demonstrates that 49% of amniocentesis testing was unnecessary and was not based on a primary infection during pregnancy [48]. Such testing is expensive and brings unnecessary risk to the unborn and anxiety to parents. Ongoing education for gynecologists should help to eliminate this unnecessary cost and risk.

In sum, while the Austrian prenatal screening protocol to minimize the effects of primary infections of *T*. *gondii* during pregnancy is cost-saving, additional cost saving could be achieved by enhancing the education of obstetric staff. There is a need to increase the number of susceptible women who receive the recommended number of screening blood tests at the recommended intervals. There is also a need to use amniocentesis only when indicated by proven primary infection during pregnancy.

### Challenges facing prevention programs

Successful screening and treatment programs, such as Austria’s, face two challenges, both of which derive from their success. As with other public health programs, the European prenatal screening programs and education campaigns confront the paradox of success. People do not see or hear about infants affected by CT as they did in the past when infant deaths or profound brain injuries and visual impairment of varying degrees were more common, due to high rates of CT. Prevention programs only seem expensive in the absence of disease. In the face of budget pressure, the absence of infants with injuries of CT can be misunderstood to mean there is no longer a risk. On the contrary, it has taken two decades of successful prenatal screening and treatment to make the risk invisible. Moreover, the success of education programs in reducing prevalence in the population, while it may protect women by making them more aware of the risk of eating undercooked meat and unwashed fruits and vegetables, actually creates a larger population of women still at risk of infection, and particularly so since even the water supply is a source of infection in some regions.

The second challenge to the prenatal screening programs comes from the methodological debate over the validity of observational studies versus randomized controlled trials as the evidence base for interventions. Numerous authors have suggested that the question of efficacy of prenatal screening and treatment can only be adequately answered with randomized controlled trials (RCTs) [13,39,66,67]. RCTs, however, pose an insurmountable ethical problem in countries where prenatal screening has been associated with significant improvement in outcomes for infants whose mothers were treated prenatally. An RCT requires equipoise, which is lacking in countries with successful screening programs (Austria, France, and Slovenia, for example) and in countries with similar epidemiology and access to care. Without equipoise, it is doubtful that one could construct an ethical trial that would require random assignment of some pregnant women to denial of a treatment with demonstrated efficacy [6,7]. Blinding could be incompatible with informed consent. It is also unlikely that such trials would have sufficient power because, with informed consent, few parents would be likely to choose not to medicate. The resulting selection bias would also invalidate the results of the trial. This ethical question is not unique to prenatal screening programs for CT. Interventions to reduce smoking, for example, were implemented based on observational data. Any RCT assigning participants to smoking would not have passed ethical review. It has been impossible to construct valid RCTs for treating sexually transmitted diseases to reduce HIV incidence because observational studies and an earlier trial demonstrated that such treatment is beneficial [68]. Similarly, any other effective treatments for cofactor infections cannot ethically be withheld from controls [68,69]. Observational and historical data from Austria, France, and Slovenia, and perhaps even comparative data from the United States, have eliminated the equipoise necessary for an ethical RCT of prenatal screening and treatment for primary infection of *T*. *gondii*. The European screening programs for CT have had noteworthy success, reducing the number of deaths and profound injuries in affected infants. That success itself in reducing preventable suffering and death commends the programs for continuation. The cost savings for national health care systems and society at large reinforce the argument for continuation.

CT is a health problem worldwide and it is not possible to eliminate all sources of infection for pregnant women, nor is a vaccine likely to be developed in the near future. There are, however, successful CT-prevention programs that are reducing clinical effects of CT and saving money for national health administrations and cost to society.

### Limitations

Our results understate the benefits of following the Austrian national program because the costs associated with injuries to infants whose mothers were not tested in accordance with the protocol are attributed to the screening scenario [31]. If those mothers had been tested on schedule, the injuries in the infants would most likely have been fewer and less severe, as was the case for the infants tested on schedule. Another source of overstatement of costs of actual Austrian practice is that we show the direct costs of ideal compliance with the protocol in obstetric visits, including the cost for all susceptible women having five tests, whereas, in practice, 97% of women had fewer than three tests. With fewer tests, that also means shorter treatment and lower treatment costs than the ideal. The average time between tests was 14 weeks, rather than the prescribed eight weeks. For two women whose infants were profoundly affected, the time between tests was 19 weeks [31]. If Austrian practice were in full compliance with the protocol, actual direct costs of screening and prenatal treatment would have been slightly higher, but the costs of treatment and accommodation of infants injured by CT and the loss of their productivity and that of their parents would have been substantially lower because fewer infants would have slipped through the screening process. The costs of screening and preventive treatment are negligible compared to the costs of treatment and accommodation for infants whose injuries are not prevented. Net benefits strongly favor screening.

### Conclusion

As demonstrated by the Austrian national program, prenatal screening and treatment result in substantial cost saving, both from the conventional societal perspective and even from the narrow perspective of budgetary impact. Results in both cases are robust to wide variations in parameter values. Our data show the positive economic value of such a prevention measure. In summary, our findings of this economic analytic-decision model represent an important base for the discussion regarding implementation or continuation of prenatal screening for toxoplasma infection.

## Supporting information

S1 Methods Explanation of decision tree, clinical variables, and costs, with detailed identification of sources. (PDF)
