# Supplementary material for: Congenital toxoplasmosis in Austria: Prenatal screening for prevention is cost-saving
Source: PLoS Negl Trop Dis. 2017 Jul 10;11(7):e0005648. doi: 10.1371/journal.pntd.0005648 (PMC5503164; doi:10.1371/journal.pntd.0005648)
Supplement: S1 Methods — (PDF) [file pntd.0005648.s001.pdf]

S1 Methods. Explanation of decision tree, clinical variables, and costs, with detailed identification of sources.

Prusa AR, Kasper DC, Sawers L, Walter E, Hayde M, and Stillwaggon E. Congenital toxoplasmosis in Austria: Prenatal screening for prevention is cost-saving.

This decision analysis finds the lower-cost or cost-saving option. It is essentially a benefit-cost analysis in which the benefits are the savings to society and to the government budget.

Part A: Probability and cost variables in decision tree

Each node in the tree is numbered as {number} to guide the reader through the tree. Node numbers are indicated in Table 1, column 1, in the article.

{1} Decision: No screening v. Screening. In the No Screening arm, all infections and injuries are discovered at birth or in infancy, except fetal death. It is assumed that there is no prenatal screening or there is haphazard screening. All infants with CT, however, are treated; society takes on the costs of accommodating injuries, such as special schooling, and bears the societal costs of lost productivity.

{2} Risk of primary infections in mothers, taken from the Austrian Toxoplasmosis Register, 1992–2008 [1].

{3} Risk of mother-to-child transmission, taken from Austrian data, almost identical to the international estimate used in the US study [1,2]. The range of estimates considered in the US study was 0.36 to 0.61, and the intermediate value of 0.50 was used. In the present work, we are evaluating the actual Austrian experience. In untreated women in Austria, the rate of congenital transmission was 0.508, which is appropriate for the No-screening scenario.

{4, 5, 7, 10, 13} The probabilities are taken from the US study [2], which incorporated all the relevant international data (European and US). There are insufficient data from the Austrian experience on untreated children; the decades of data from other sources give us a better picture of the outcomes for untreated mothers and children.

{6} A child who has no symptoms (under the no screening scenario) will generate no costs beyond the usual well-baby care.

{8, 9, 11, 12, 14} These costs reflect the costs of monitoring and treating a symptomatic child, based on Austrian practice and estimates. They are net of routine well-baby care, for example routine visits in the first year.

{8} **Visual mild = VisualMild**

For mild visual disability, we assume no productivity loss for affected children or their parents.

The derivation of cost estimates is explained below in Part B.

{9} **Visual severe = VisualMild + VisualSevere + SpecEdBlind**

For children with severe visual disability, we use treatment costs for mild visual impairment, VisualMild, plus non-medical direct costs and productivity losses derived from Lafuma *et al.* [3], which includes child income loss and parent productivity loss.

{11} **Visual and cognitive mild = VisualMild + CognMild + SpecEdMildCogn**

{12} **Visual and cognitive severe = VisualMild + CognSevere + SpecEd SevereCogn + ParentPyLoss + ChildPyLoss**

In order not to double count home care, parent productivity loss, and other non-medical costs for a child with severe visual and cognitive disability we use treatment costs for mild visual disability. Severe cognitive disability entails a child productivity loss, that is, the child is unable to work upon reaching working age, and a parent productivity loss, neither of which is included in Gustavsson *et al.* [4] costs for "Mental retardation," which is used to calculate CognSevere.

{14} **Visual, cognitive, and hearing severe = VisualMild + CognSevere + HearingMild + SpecEd SevereCogn + ParentPyLoss + ChildPyLoss**

{15}, {26}, {38}, {49}, {62}, {73} **Fetal or neonatal death = VSL**

The cost of a fetal or neonatal death is the Value of a Statistical Life, discussed below in Part C.

{16, 17} No costs result for these outcomes.

{18} If all women are screened at 8 weeks gestation, then 34% (0.344) will be IgG+, based on Austrian prevalence.

{19} Of these IgG+ women, most will have been infected before pregnancy, and 0.000845 of women will have new infections (IgM+).

{20, 21} Because there were so few cases in the Austrian data, the international estimate is used [2].

{22 to 26} For each possible outcome, the costs are listed at the terminal node (the triangle). The pattern is the same throughout. For example, at {22}, for a child with CT whose mother's serology indicates that she was infected during the first 8 weeks of gestation and who is asymptomatic, the costs will be 1 maternal IgG test, 1 maternal IgM test, 5 infant IgG tests, 5 infant IgM tests, pediatric treatment for a year, pediatric CBC, one ECG, one cranial ultrasound, 17 funduscopies beyond routine care, and the cost of maternal treatment when CT is identified (RxPosPCR). In {23 to 26} there are additional costs due to symptoms, including death {26}.

{23 to 74} Loss of child productivity is not included in the screening (with pre- and post-natal treatment) arm because, in Austria, in all cases the children are able to work upon reaching working age. The Austrian Toxoplasmosis Register [1] records the specific effects in each child, so each node in the screening branch is tailored to actual experience of the children affected.

{23}, {33}, {44}, {55}, {70} **Visual = VisualMild**

Visual impairment is mild in all children who are treated.

{24} **Visual and cognitive mild = VisualMild + CognMild**

Visual and cognitive impairment is mild in all children who are treated.

{25} **Visual, cognitive & hearing with cerebral CT = VisualMild + CognMild + HearingMild + Cerebral**

{27} In this branch, there is no CT. The mother is treated for the No CT case (RxNegPCR), that is, with a negative PCR of amniotic fluid. The child is monitored for the first year and has one funduscopy at birth. There is no ECG (ECG is needed before treating infants with spiramycin and these infants will not be treated).

{28} When the mother is found to have been infected before pregnancy, the only cost is for maternal IgG and IgM. Note that in the rest of the tree, there are no maternal IgM tests because only at the 8-week test is there uncertainty about whether the maternal infection occurred during pregnancy. In actual cases, not all mothers are tested at 8 weeks. If their first test occurs at 16 weeks, they might have an IgM test, but there is no way to include that detail of non-compliance in the model. The screening costs are trivial and unimportant for determining the lower-cost outcome. The potential injuries to the child from delayed testing, however, are very important. As we note in the article, the model both understates and overstates cost of best practice (full compliance) in two ways: 1) not as many screenings are conducted as we include in the tree (overstating actual costs of screening, but understating costs of treatment for mothers identified with primary infections), and 2) children suffer injuries that might have been avoided with better compliance, and those injuries entail costs that we do include (overstating costs of injuries if compliance had been optimal).

{29} Women who are IgG– at 8 weeks are retested and 0.000845 of them will have primary infections.

{30} Risk of fetal infection based on Austrian Toxoplasmosis Register, 36/217 = 0.17.

{31, 34} Based on the Austrian Toxoplasmosis Register.

{32, 33, 35, 36, 37, 38} These costs are based on Austrian protocol and specific outcomes are based on the Austrian Toxoplasmosis Register.

{35} **Visual and cognitive with cerebral CT and special schooling = VisualMild + CognMild + Cerebral + SpecEdMildCogn**

{36} **Visual and cognitive with cerebral CT and special schooling = VisualMild + CognMild + Cerebral**

The children at nodes 35 and 36 had cerebral CT, but not all required special schooling.

{37}, {48}, {61}, {72} **Visual, cognitive, and hearing = VisualMild + CognMild + HearingMild**

{39} Only one funduscopy at birth is administered because there is no CT.

{40} Women negative at 16 weeks are retested at 24 weeks, of whom 0.000845 have primary infections.

{41} Based on Austrian Toxoplasmosis Register, 36/398.

{42, 45} Based on Austrian Toxoplasmosis Register.

{43, 44, 46, 47, 48, 49, 50} Based on Austrian costs and Austrian Toxoplasmosis Register.

{46} **Cognitive with cerebral CT = CognMild + Cerebral**

{47} **Visual with physical = VisualMild +CognMild**

The children at nodes 46 and 47 have mild cerebral CT or mild visual and physical impairment. None of the children in the treatment arm is unable to work.

{51} Women negative at 24 weeks are retested at 32 weeks, with 0.000845 incidence.

{52} Based on Austrian Toxoplasmosis Register, 48/368

{53, 56, 57} Based on Austrian Register

{54, 55, 58, 59, 60, 61, 62} Based on Austrian protocol and costs

{58} **Cerebral CT with special schooling = CognMild + Cerebral + SpecEdMildCogn**

{59} **Cerebral CT with no special schooling = CognMild + Cerebral**

{60} **Visual with physical impairment = VisualMild + CognMild**

{63} When the mother is infected late in pregnancy, there is a high risk of fetal infection. Fetal infection was ruled out through PCR in 57% of cases, and the children were not treated. For late-infected women without PCR (43%), their children were treated for one year and had a year of CBC.

{64} **High risk of fetal infection with no PCR =** Children are treated as a precaution.

{65} **High risk of fetal infection with negative PCR =** Children are not treated.

{66} All seronegative or unscreened mothers are tested at birth, or the newborn is tested. For simplicity, both are designated as maternal test, with the same primary infection rate of 0.000845.

{67} Based on the Austrian Toxoplasmosis Register, 18/49 were seropositive, or 0.37.

{68} Based on the Austrian Toxoplasmosis Register.

{69–74} Based on the Austrian Toxoplasmosis Register, the protocol, and Austrian costs

{71} **Cerebral CT = CognMild + Cerebral**

{74} The mother is not treated because the test is at the time of birth. The baby is treated for one year because of high risk of transmission and has one funduscopy.

{75} The only cost for the great majority of mothers is for the 5 IgG tests. (The number of women who remain $IgG^-$is 1,387,680 minus 1173 primary infections.)

Part B: Derivation of costs used at terminal nodes in decision tree

Sources for the first 12 items in Table 2 are listed in the table. The following explains derivations of treatment and accommodation costs based on levels of disability and productivity losses listed in Table 2. When Austrian costs were not available, we used costs from nearby countries with similar health systems and adjusted to Austrian costs as explained below.

Cerebral CT (treatment cost, years 0 to 4) is based on the costs for "Epilepsy" from Gustavsson *et al.* [4] Table 7, page 730. They estimated the annual cost of epilepsy per person in Austria in 2010 to be €6,079. We adjust Austrian costs from 2010, when the consumer price index was 109.53 to 2012 when the consumer price index was 116.34 (Eurostat, "HICP (2005 = 100) – annual data (average index and rate of change)," http://ec.europa.eu/eurostat/en/web/products-datasets/-/PRC_HICP_AIND) [5]. The ratio is 1.062, that is, consumer prices rose 6.2% in Austria between 2010 and 2012. Adjusting to Austrian prices in 2012, the annual cost is

€6,079 x 1.062 = €6,457.

Since these children have relatively mild outcomes and their symptoms are controlled, we apply these costs only for 5 years. Total cost for 5 years discounted at 3% annually is €30,458. Our analysis estimates the value of a stream of economic costs into the future. Future costs are assumed to be worth less than current ones, so costs and benefits in the future must be weighted less than those in the near term to

determine what economists call the present discounted value. The annual rate of discount most commonly used in health economics is 3% [6].

Child productivity loss is the life-time wage loss due to severe impairment that prevents gainful employment. The OECD reports the mean Austrian annual wage for full-time, full-year employees for 2012 to be €38,273, (OECD.Stat, "Average Annual Wage," https://stats.oecd.org/Index.aspx?DataSetCode=AV_AN_WAGE) [7]. The mean retirement age in Austria reported by the OECD ("Ageing and Employment Policies – Statistics on average effective age of retirement," http://www.oecd.org/els/emp/ageingandemploymentpolicies-statisticsonaverageeffectiveageofretirement.htm) [8] is 60.5 years. The total wage loss from age 18 to age 60.5 discounted at 3% annually is €561,721.

Cognitive mild (direct and indirect costs) is derived from Gustavsson *et al.* [4] Table 11, page 734, which gives annual cost per person as weighted means for all diagnoses and age groups within the disorder. For this category we use "Child and adolescent disorders," which covers several developmental disorders, and includes direct and indirect costs. Indirect costs for this category include parent productivity loss. Gustavsson *et al.* estimate the annual cost of child and adolescent disorders for Austria in 2010 to be €4,391. We adjust to 2012 prices,

€4,391 x 1.062 = €4,664.

The total cost of mild cognitive impairment over 18 years discounted at 3% annually is €66,071.

Cognitive severe (direct costs and some indirect costs, lifetime) is based on Gustavsson *et al.* [4] Table 11, page 734. Their estimate of the cost of "Mental retardation" includes direct and some indirect costs but does not include lifetime productivity loss for the parent or for the child upon reaching working age. For Austria in 2010, Gustavsson *et al*. estimate an annual cost of €13,404. Adjusting to 2012 prices, annual cost of mental retardation in 2012 in Austria is

€13,404 x 1.062 = €14,237.

Life expectancy in Austria is 80.5 years (OECD, "Life expectancy has increased remarkably in OECD countries," www.oecd.org/berlin/47570143.pdf) [9]. The lifetime costs of mental retardation discounted at 3% annually are €445,536.

Hearing mild (treatment cost, lifetime) Hearing loss is not seen in children with CT in the absence of other outcomes. Even though hearing loss might be profound, we estimate the cost only for mild hearing loss (correctable with hearing aid) in order to avoid double counting for special schooling, parental productivity loss, and other treatment and indirect costs. Thus we include only the cost of hearing aids, which in Belgium, without subsidies, is €3,500 (Hear-it, at http://www.hear-it.org/hearing-loss-Belgium.) Hearing aids need to be replaced every five years (Emory University, http://www.emoryhealthcare.org/ear-nose-throat/audiology/faq-hearing-aids.html, and Hear-it, http://www.hear-it.org/hearing-loss-Denmark). We do not include the cost of batteries or hearing tests. We adjust for the difference in consumer price levels between Belgium and Austria in 2012 using Eurostat, "Comparative price levels of final consumption by private households including indirect taxes (EU 28 = 100)," (http://ec.europa.eu/eurostat/tgm/printTable.do?tab=table&plugin=1&language=en&pcode=tec00120&printPreview=true) [10]. Adjusting to the Austrian price level, the cost in 2012 is:

€3500 x 0.0962 = €3,366.

The total cost for hearing aids replaced every five years beginning at age 2 until age 77 discounted at 3% annually is €20,924.

Parent productivity loss (years 0 to 18) is based on estimates of mother's work reduction from Lange *et al.* [11] Tables 1 and 2, page 1131, calculated in Walter *et al.* [12] Table 7.9, page 134. Walter *et al.* estimate the mother's annual earnings loss for Germany in 2008, for children < 6 years, 6 to 10 years, and 11 to 18 years. We adjust from German to Austrian prices for 2008 using Eurostat, "Comparative price levels of final consumption by private households including indirect taxes (EU 28 = 100)" [10]. Austrian prices were 1.0154 higher than German prices in 2008. We adjust Austrian costs to the price level in 2012 using Eurostat, "HICP (2005 = 100) - annual data (average index and rate of change)" [5]. Austrian prices increased 8.45% between 2008 and 2012.

We adjust each of the annual earnings loss estimates for Germany in 2008 to Austrian prices in 2012, as follows:

< 6 years:	€3,418.58 x 1.0154 x 1.0845 = €3,764.54
6 to 10 y:	€2,084.36 x 1.0154 x 1.0845 = €2,295.30
11 to 18 y:	€ 672.34 x 1.0154 x 1.0845 = € 740.38

The total parental earnings loss over 18 years discounted at 3% annually is €33,940.

Special Ed, severe visual impairment is based on a study by Walter *et al.* [12] Table 7.9, page 134. They estimate the cost of schooling for blind children in Germany in 2008 to be €10,333 per year for 10 years. We adjust that amount to Austrian prices in 2012 using Eurostat, "Comparative price levels of final consumption by private households including indirect taxes (EU 28 = 100)" [10] and "HICP (2005 = 100) - annual data (average index and rate of change)" [5], for an estimate of annual per-child cost of special schooling of

€10,333 x 1.0154 x 1.0845 = €11,379.

Assuming children have special schooling from age 5 to 14, total schooling cost discounted at 3% annually is €86,239.

Special Ed, mild cognitive impairment is based on a study by Walter *et al.* [12] Table 7.9, page 134. They estimate annual costs of schooling in Germany in 2008 for those with mild cognitive impairment to be €6,126. We adjust German schooling costs in 2008 to Austrian prices in 2012 using Eurostat, "Comparative price levels of final consumption by private households including indirect taxes (EU 28 = 100)" [10] and "HICP (2005 = 100) - annual data (average index and rate of change)" [5], as follows:

€6,126 x 1.0154 x 1.0845 = €6,746.

The children receive 15 years of special schooling from age 4 to 18. Total costs are discounted at 3% annually and amount to €73,699.

Special Ed, severe cognitive impairment is based on a study by Walter *et al.* [12] Table 7.9, page 134. They estimate the cost of schooling for children with severe cognitive impairment in Germany in 2008 to be €44,835 per year for 20 years. We adjust that amount to Austrian prices in 2012 using Eurostat, "Comparative price levels of final consumption by private households including indirect taxes (EU 28 = 100)" [10] and "HICP (2005 = 100) - annual data (average index and rate of change)" [5], for an estimate of annual per-child cost of special schooling of

€44,835 x 1.0154 x 1.0845 = €49,372.

Assuming the children receive special schooling from age 2 to 21, total costs for 20 years discounted at 3% annually amount to €713,141.

Visual mild (treatment cost only, age 0 to 18) is based on a study by Walter *et al*. [12] Table 7.6 and 7.7, pages 130–131. They give estimates of treatment costs for visual impairment in children with CMV who were either symptomatic or asymptomatic at birth, assuming a 5% annual discount rate. Averaging those two estimates gives the following treatment costs of visual impairment as

€1,258 + €1,222 = €2,480/2 = €1,240.

To determine the treatment costs with the 3% annual discount rate that is used throughout our study, we calculate the annual cost of treatment, assuming that costs per year are uniform from age 0 to 18, using the following formula

$$\text{Annual cost} = [1240\,(1+r)^{Y-1}r\,] / [\,(1+r)^{Y} - 1],$$

where r is the annual discount rate and Y is the year.

The annual treatment cost that would produce a total cost of €1,240 for 18 years discounted at 5% is €100.03. The formula for deriving the annual expenditure from a discounted present value can be found at moneychimp (http://www.moneychimp.com/articles/finworks/fmpayout.htm), which also provides an annuity calculator (http://www.moneychimp.com/calculator/annuity_calculator.htm).

We adjust the German costs in 2008 to Austrian prices in 2012 using Eurostat, "Comparative price levels of final consumption by private households including indirect taxes (EU 28 = 100)" [10] and "HICP (2005 = 100) - annual data (average index and rate of change)" [5], producing the following estimate of annual treatment costs in Austria:

€100.03 x 1.0154 x 1.0845 = €111.25.

Total Austrian treatment cost over 18 years discounted at 3% annually is €1,576.

Visual severe (non-medical costs) is composed of two separate costs, the loss of earnings from severe visual impairment and other non-medical costs associated with severe visual impairment. These costs must be calculated separately since they occur at different periods over a person's lifetime. Estimates of both costs are based on a study of visual impairment in four European countries by Lafuma *et al*. [3]. In Table II, page 199, they report estimated annual per person income loss from severe visual impairment in Germany in 2004 to be

€3,705.14 + €137.23 = €3,842.

The calculation of lifetime earnings loss from severe visual impairment assumes people work from age 18 to the mean retirement age of 60.5 in Austria (OECD, "Ageing and Employment Policies – Statistics on average effective age of retirement") [8]. We adjust average annual German income loss in 2004 to Austrian prices in 2012 using Eurostat, "Comparative price levels of final consumption by private households including indirect taxes (EU 28 = 100)" [10] and "HICP (2005 = 100) - annual data (average index and rate of change)" [5], and find annual earnings loss from severe visual impairment in Austria in 2012 to be

€3,842.37 x 0.9857 x 1.18787 = €4,499.

Total income loss for ages 18 to 60.5 discounted at 3% annually is €66,030.

Again using Lafuma *et al*. [3] Table II, page 199, we determine the costs of severe visual disability other than loss of earnings by subtracting the cost of lost earnings from total annual, per person societal costs (including unmet needs)

€12,783.04 – €3,842.37 = €8,940.67.

We adjust annual German costs in 2004 of disability from severe visual impairment excluding income loss to estimate Austrian costs in 2012 using Eurostat, "Comparative price levels of final consumption by private households including indirect taxes (EU 28 = 100)" [10] and "HICP (2005 = 100) - annual data (average index and rate of change)" [5]. Non-medical costs arising from severe visual impairment excluding earnings loss in Austria in 2012 are thus

€8,940.67 x 0.9857 x 1.18787 = €10,468.

Total other non-medical costs excluding earnings loss for all persons with severe visual impairment age 0 to 80.5, the expected life-span of Austrians (OECD, "Life expectancy has increased remarkably in OECD countries" [9]) discounted at 3% annually are €327,594.

Total non-medical costs (including earnings loss during working years) are the sum of the two figures calculated above, the loss of earnings from age 18 to 60.5 and other non-medical costs incurred over the lifetime:

€66,030 + €327,594 = €393,624.

Part C: Value of a Statistical Life

Measures of the value of a statistical life (VSL) are routinely used in the economic evaluation of public policies that may lead to higher or lower mortality. As a recent publication of the Organization of European Cooperation and Development (OECD) put it, "policy makers are regularly devising policies and regulations that affect people's risk of death and that seek to protect lives in society, and require methodologies for comparing the costs of reducing risk with the expected benefits in terms of lives saved. The benefits of prevented mortalities can be expressed in terms of a 'Value of a Statistical Life' (VSL), which represents the value a given population places *ex ante* on avoiding the death of an unidentified individual" (from the Foreword of [13]).

There is a voluminous literature on measuring VSL in Europe and other industrialized countries [13-16]. Initially, the discourse over VSL typically employed a human capital approach to measuring VSL based on the estimated value of income over a lifetime. The human capital approach has been supplanted by the stated preference approach favored by most European economists and the revealed preference approach used by most US economists. The latter two methodologies produce substantially higher measures of VSL than the human capital approach.

The OECD recently assessed over 800 studies that use the stated preference approach to measuring VSL [13]. That meta-analysis found quality-screened measures of VSL ranging between US $1.8–5.4 million (in 2005 US dollars) with median and mean of US $3.6 million. The OECD recommends accepting the mean/median figure as the "base value" to be adjusted for the purposes of estimating the VSL for the particular group under consideration (page 127 in [13]). They add, "when the policy that is analysed targets children specifically (or affects mainly children), a higher VSL for children is recommended, based on the available empirical evidence from the United States and Europe. *VSL for children should be 1.5-2.0 times higher than the mean adult VSL*" (page 131 in [13], emphasis in original).

Five studies measured VSL in Austria [17-21]. In the last quarter century, substantial progress has been made in the methodology of measuring VSL, and only three of the five studies were carried out since the 1980s. Two of the recent measures of VSL in Austria lie within the OECD's range of VSL of US $1.8–5.4 million (in 2005 US dollars) [19,20], and the third is just below that range [21]. Rather than base our

statistical analysis on three studies, we opt to follow the OECD's recommendation since it is based on hundreds of studies.

We adjust OECD's recommendation for VSL, stated in US dollars in 2005, to the price level in Austria in 2012 using Eurostat, "Comparative price levels of final consumption by private households including indirect taxes (EU 28 = 100)" [10] and "HICP (2005 = 100) - annual data (average index and rate of change)" [5]. Exchange rate data are taken from OANDA (http://www.oanda.com/currency/average/).

At present, most US economists measure VSL using a revealed preference approach based on income differentials among occupations with different mortality risks. Datasets that have recently become available in the United States allow much more precise measures of income and mortality risk by occupation, industry, gender, age, and other categories. The new data lead to measures of VSL that are typically higher than both the earlier measures using the revealed preference approach and current measures using the stated preference approach, which is commonly used in Europe [22]. Recent US government benefit-cost analyses use measures of adult VSL that range between US$6 million and US$10 million (in 2013 dollars), equivalent to €4.6–€7.7 million in 2012 euros [23]. In the United States, several authors discourage upward adjustment of VSL for children [24]. In short, the US and European approaches to VSL, though they use different methodologies and different age adjustments, arrive at recommended measures of VSL that are remarkably similar (€5–€6.7 million in Europe vs. €4.6–€7.7 million in the United States).

We use productivity losses derived from the literature as explained in Part B for surviving children with vision, hearing, or cognitive impairment. None of those estimates is adequate for attributing a value to fetal or neonatal death, which is why we use VSL in those few cases [25].

References

1. Prusa AR, Kasper DC, Pollak A, Gleiss A, Waldhoer T, et al. (2015) The Austrian Toxoplasmosis Register, 1992-2008. Clin Infect Dis 60: e4-e10.
2. Stillwaggon E, Carrier CS, Sautter M, McLeod R (2011) Maternal serologic screening to prevent congenital toxoplasmosis: a decision-analytic economic model. PLoS Negl Trop Dis 5: e1333.
3. Lafuma A, Brezin A, Lopatriello S, Hieke K, Hutchinson J, et al. (2006) Evaluation of non-medical costs associated with visual impairment in four European countries: France, Italy, Germany and the UK. Pharmacoeconomics 24: 193-205.
4. Gustavsson A, Svensson M, Jacobi F, Allgulander C, Alonso J, et al. (2011) Cost of disorders of the brain in Europe 2010. European Journal of Neuropsychopharmacology 21: 718-779.
5. Eurostat (2015) HICP (2005 = 100) – annual data (average index and rate of change). Luxembourg: European Commission.
6. Corso PS, Haddix AC (2003) Time Effects. In: Haddix AC, Teutsch SM, Corso PS, editors. Prevention Effectiveness: A Guide to Decision Analysis and Economic Evaluation. 2nd ed. New York: Oxford University Press. pp. 92-102.
7. OECD (2015) Average Annual Wage. Paris: Organization for European Co-operation and Development.
8. OECD (2015) Ageing and Employment Policies – Statistics on average effective age of retirement. . Paris: Organization for European Co-operation and Development.
9. OECD (2015) Life expectancy has increased remarkably in OECD countries. Paris: Organization for European Co-operation and Development.
10. Eurostat (2015) Comparative price levels of final consumption by private households including indirect taxes (EU 28 = 100). Luxembourg: European Commission.
11. Lange K, Danne T, Kordonouri O, Berndt V, Muller B, et al. (2004) Diabetesmanifestation im Kindesalter: Alltagsbelastungen und berufliche Entwicklung der Eltern [Diabetes in childhood:

everyday burden and professional consequences for parents]. Deutsche medizinische Wochenschrift 129: 1130-1134.
12. Walter E, Brennig C, Schöllbauer V (2011) How to save money: congenital CMV infection and the economy. In: Halwachs-Baumann G, editor. Congenital Cytomegalovirus Infection. Vienna: Springer. pp. 121-144.
13. OECD (Organization for European Co-operation and Development) (2012) Mortality Risk Valuation in Environment, Health and Transport Policies. Paris: OECD Publishing.
14. de Blaeij A, Florax RJ, Rietveld P, Verhoef E (2003) The value of statistical life in road safety: a meta-analysis. Accid Anal Prev 35: 973-986.
15. Viscusi WK, Aldy JE (2002) The value of a statistical life: critical review of market estimates throughout the world, Discussion Paper No. 392. Cambridge, MA: Harvard Law School
16. Miller TR (2000) Variations between Countries in Values of Statistical Life. Journal of Transport Economics and Policy 34: 169-188.
17. Weiss P, Maier G, Gerking S (1986) The economic evaluation of job safety: a methodological survey and some estimates for Austria. Empirica - Austrian Economic Papers 13: 53-67.
18. Maier G, Gerking S, Weiss P (1989) The economics of traffic accidents on Austrian roads: risk lovers or policy deficit? Empirica- Austrian Economic Papers 16: 177-192.
19. Leiter A, Pruckner G (2008) Dying in an avalanche: current risks and their valuation. University of Innsbruck, Working Paper 2008-11: Innsbruck.
20. Leiter A, Pruckner G (2009) Proportionality of willingness to pay to small changes in risk: the impact of attitudinal factors in scope tests. Environmental and Resource Economics 42: 169-186.
21. Leiter AM, Pruckner GJ (2014) Timing effects in health valuations. Health Econ 23: 743-750.
22. Viscusi WK, Zeckhauser RJ (2015) The relative weights of direct and indirect experiences in the formation of environmental risk beliefs. Risk Anal 35: 318-331.
23. Robinson LA (2007) How US Government Agencies Value Mortality Risk Reductions. Review of Environmental Economics and Policy 1: 283–299.
24. Dockins C, Maguire K, Simon N, Sullivan M (2004) Value of Statistical Life Analysis and Environmental Policy: A White Paper. U.S. Environmental Protection Agency, National Center for Environmental Economics, Washington D.C.
25. Haddix AC, Teutsch SM, Corso PS, editors (2003) Prevention Effectiveness: A Guide to Decision Analysis and Economic Evaluation. 2nd ed. New York: Oxford University Press.
